# Mugwort-Mustard Allergy Syndrome due to Broccoli Consumption

**DOI:** 10.1155/2016/8413767

**Published:** 2016-07-13

**Authors:** Yuri Sugita, Teruhiko Makino, Megumi Mizawa, Tadamichi Shimizu

**Affiliations:** Department of Dermatology, Graduate School of Medicine and Pharmaceutical Sciences, University of Toyama, Toyama 930-0194, Japan

## Abstract

Pollen-food allergy syndrome (PFAS) is a relatively rare form of food allergy which develops in individuals who are sensitized to pollen. Tree pollens, especially birch pollen, frequently induce PFAS; however, the incidence of PFAS due to grass or weed pollens such as ragweed or mugwort is relatively rare. Mugwort-mustard allergy syndrome (MMAS) is an example of a PFAS in which individuals sensitized to mugwort may develop an allergy to mustard and experience severe reactions. We herein describe a case of MMAS due to broccoli consumption.

## 1. Introduction

Pollen-food allergy syndrome (PFAS) is a relatively rare form of food allergy which develops in individuals who are sensitized to pollen [1]. Tree pollens, especially birch pollen, frequently induce PFAS; however, the incidence of PFAS due to grass or weed pollens, such as ragweed or mugwort is relatively rare. Mugwort-mustard allergy syndrome (MMAS) is an example of a PFAS in which individuals sensitized to mugwort may develop an allergy to mustard and experience severe reactions [2]. We herein describe a case of MMAS due to broccoli consumption in a Japanese male.

## 2. Case Report

**Figure 1:**
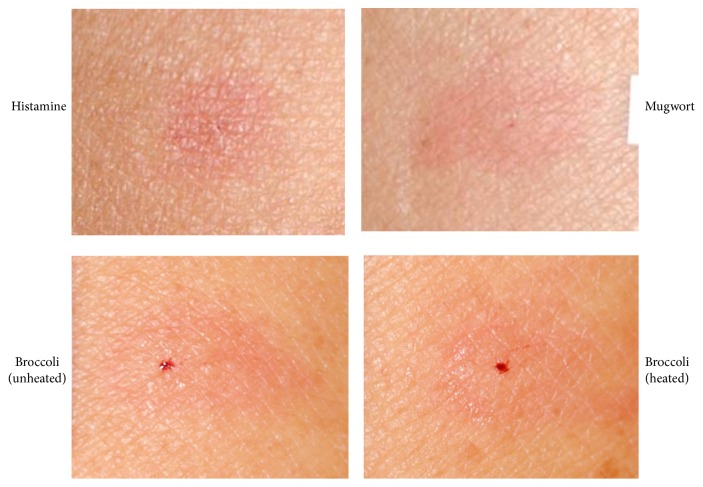
A skin-prick test with mugwort, raw broccoli, and heated broccoli showed wheal and erythema.

A 73-year-old Japanese male presented to our hospital with dyspnea and swelling of the lips and eyelids which occurred 30 minutes after eating boiled broccoli. He had no history of allergic reactions to latex or fruits. The patient's clinical findings suggested he was experiencing an immediate-type hypersensitivity reaction to broccoli, such as oral allergy syndrome (OAS) or PFAS. Therefore, tests for allergic reactions including the CAP fluorescent enzyme immunoassay RAST (CAP-FEIA RAST) test and a skin-prick test (SPT) were performed. A blood test revealed an elevated level of serum immunoglobulin E (IgE: 681 IU/mL; normal range: 0–170 IU/mL) and IgE antibodies specific for mugwort (0.83 UA/mL; normal range: 0–0.34 UA/mL) were detected in the CAP-FEIA RAST test, although no IgE antibodies specific for almond or mustard were detected. An SPT was then performed using the prick-to-prick technique with samples of mugwort, broccoli, cauliflower, cabbage, mustard, almond, and peanut. The results of the SPT were assessed as false positive (+), positive (2+), strongly positive (3+), and most positive (4+) when the average wheal diameter was more than 25%, 50%, 100%, and 200%, respectively, of a positive control induced by 10 mg/mL of histamine dihydrochloride 30 minutes after administration [3]. Positive reactions were observed for mugwort (3+), raw broccoli (4+), and mustard (3+) (Figure 1). Heated broccoli (4+) and heated mustard (3+) also showed positive reactions. The test was negative for cauliflower, cabbage, almonds, and peanuts. Based on the clinical symptoms and the results of the allergy tests, the patient was diagnosed with MMAS due to broccoli consumption. The patient was therefore advised to avoid consuming broccoli and mustard. He has not experienced any symptoms of MMAS since.

## 3. Discussion

Mugwort pollens are known to cross-react with some fruits and vegetables. Therefore, mugwort-related PFAS is classified as celery-mugwort-spice syndrome (CMSS), MMAS, mugwort-peach association (MPA), or mugwort-chamomile association (MCA) [4]. Patients experiencing MMAS who are sensitized to mugwort commonly develop clinical symptoms such as pruritus, erythema, and subtle angioedema following ingestion of mustard. Additionally, MMAS is known to occasionally cause severe systemic reactions, including anaphylaxis. Figueroa et al. demonstrated that 97% of 38 patients with mustard allergy had already been sensitized to mugwort pollen and that all of these patients were allergic to other foods belonging to the Brassicaceae family, such as cauliflower and cabbage. Only one individual has so far been reported to develop an allergy to broccoli, although the details of the clinical findings were unclear [2]. Recently, Dölle et al. reported a case of broccoli-induced anaphylaxis, which might have been caused by the inhalation of pollen allergens, including grass, mugwort, and ambrosia [5]. In this case, the clinical symptoms appeared to include MMAS, MPA, and MCA. Furthermore, the cross-reactive allergen was suspected to be Pru P3, the peach lipid transfer protein (LTP), or Art v3, the mugwort LTP. In contrast, the case presented herein had elevated levels of IgE antibodies for mugwort and the SPT indicated a positive reaction for only broccoli and mustard. Therefore, we believe that our patient exhibited the typical symptoms of MMAS. To the best of our knowledge, there are no published reports of a case of MMAS due to broccoli ingestion.

Pollen allergens of MMAS are believed to include Art v4 (a profilin), Art v3 (an LTP), and Art v60 kDa (a high-molecular weight allergen) [4–6]. LTPs are reported to be thermostable and resistant to peptic digestion. Therefore, they cause not only OAS but also severe systemic reactions. LTPs have been identified as a major protein on the surface of broccoli leaves [7]. We speculate that the causative food allergen in the present case might be related to LTPs in broccoli, because the patient exhibited symptoms of OAS and swelling of the eyelids and dyspnea after consuming heated broccoli. Additionally, the SPT showed a positive reaction for heated broccoli.

Further studies are needed to identify the causative allergens of MMAS because the cross-reactivity between Art v3 and LTP in broccoli remains a possibility. However, the present case will help expand our knowledge of MMAS.
